# Unproven stem cell interventions: A global public health problem requiring global deliberation

**DOI:** 10.1016/j.stemcr.2021.05.004

**Published:** 2021-06-08

**Authors:** Zubin Master, Kirstin R.W. Matthews, Mohamed Abou-el-Enein

**Affiliations:** 1 Biomedical Ethics Research Program and the Center for Regenerative Medicine, Mayo Clinic, Rochester, MN, USA; 2 Baker Institute for Public Policy Center for Health and Biosciences, Rice University, Houston, TX, USA; 3 Division of Medical Oncology, Department of Medicine, and Department of Stem Cell Biology and Regenerative Medicine, Keck School of Medicine, University of Southern California, Los Angeles, CA, USA; 4 Joint USC/CHLA Cell Therapy Program, University of Southern California, and Children Hospital Los Angeles, Los Angeles, CA, USA

**Keywords:** unproven stem cell interventions, global health, regenerative medicine, regulations, patient views, World Health Organization

## Abstract

The unproven stem cell intervention (SCI) industry is a global health problem. Despite efforts of some nations, the industry continues to flourish. In this paper, we call for a global approach and the establishment of a World Health Organization (WHO) Expert Advisory Committee on Regenerative Medicine to tackle this issue and provide guidance. The WHO committee can harmonize national regulations; promote regulatory approaches responsive to unmet patient needs; and formulate an education campaign against misinformation. Fostering an international dialog and developing recommendations that can be adopted by member states would effectively address the global market of unproven SCIs.

## Teaser

In this article, Master, Matthews, and Abou-el-Enein argue that the World Health Organization (WHO) should establish an Expert Advisory Committee on Regenerative Medicine to address the global problem of clinics selling unproven stem cell interventions (SCIs). The WHO committee can develop harmonized standards, promote responsible access to cell-based therapeutics for patients, and create an information campaign to address public misinformation.

## Main text

The unproven stem cell intervention (SCI) industry is used to describe a worldwide, direct-to-consumer market where clinics offer stem cells, stem cell-derived components, such as exosomes and non-stem cell-based cellular products to patients with little to no scientific or clinical basis (Turner, 2020; U.S. Food and Drug Administration, 2019). The application of unproven SCIs to consumers has led to multiple patient injuries and deaths (Bauer et al., 2018), and the industry threatens legitimate research efforts (Berger et al., 2016) and undermines regulatory authority to safeguard public health. The issue of marketing unproven SCIs spans national borders, requiring international coordination and cooperation as no one country can (or has) effectively addressed the issue by itself.

In this paper, we review the international unproven SCI industry and highlight the national efforts made in the US as a case example of a country with robust regulations but with a flourishing market for unproven SCIs. We argue that the marketing of SCIs and its associated harms have reached an international scale and become a major global health threat requiring global thinking, deliberation, and a shared understanding of norms and practices. Taking cues from the recent endeavor of the World Health Organization (WHO) to consider the ethics and practice of human genome editing, we suggest that a similar approach may be adopted to tackle the issue of the worldwide marketing of unproven SCIs. While our proposal outlines a specific initiative, our goal, however, is for the scientific community to consider the issue beyond one or a few largely independent organizations operating in one or more countries and to adopt a more global outlook to successfully tackle this problem (Lee et al., 2017; Sipp et al., 2017).

### The unproven SCI industry: A global health problem

WHO is dedicated to addressing the global burden of non-communicable diseases, including heart disease, cancer, diabetes, and respiratory illness, which are responsible for 70% of the deaths worldwide (World Health Organization, 2019a) with rising rates given the growth of the aging population (Terzic and Nelson, 2020). Regenerative medicine, a branch of research involving the generation, manipulation, and use of therapeutic (stem) cells and tissues, among other products, aims primarily to address the burden of non-communicable diseases. The regenerative medicine community consist mainly of research organizations with public and private investments constituting a global market of about US$13 billion (Regenerative Medicine Market, 2019) with over 1,200 cell and gene therapy trials, mostly in early-phase clinical research (Alliance for Regenerative Medicine, 2021). Despite the tremendous growth in the regenerative product pipeline, only a few stem cell products have emerged to date aside from bone marrow transplant-related procedures for hematologic cancers (Abou-El-Enein et al., 2016; Cossu et al., 2018; Cuende et al., 2018). As regenerative medicine aims to address the chronic disease burden worldwide, ensuring proper governance, including curtailing the unproven SCI market, is within the WHO’s purview.

The unproven SCI industry has been growing internationally and several factors may account for the rising number of clinics. The media hype suggesting that stem cell research will lead to novel cures creates a public perception that stem cell therapies are or will soon be available (Caulfield et al., 2016; Kamenova and Caulfield, 2015). Rather than undertaking the substantive investment necessary to ensure cell therapies are safe and effective through clinical research, some clinics have taken advantage of this public perception to market unproven SCIs. The idea that SCIs are available and beneficial has further spread through online platforms, including websites, blogs, and social media (Hawke et al., 2019; Kamenova et al., 2014; Marcon et al., 2017; Petersen et al., 2016, 2019). Without sufficient expert warnings surrounding unproven SCIs (Salter et al., 2015), patients and the public may have difficulty discerning between credible information from misinformation. In the name of faster access of regenerative products to patients, some countries have or are considering loosening regulatory standards, which permits an economic advantage to individual nations in a highly competitive global market but, in turn, may help flourish the unproven SCI industry (Sipp and Sleeboom-Faulkner, 2019; Sleeboom-Faulkner, 2019).

Earlier analyses indicated that unproven SCI clinics were predominately located in countries considered to have less regulatory enforcement over drugs and biologics. Since 2014, clinics marketing purported SCIs have emerged in countries with robust regulatory infrastructure within Europe, North America, Australia, and Southeast Asia (Berger et al., 2016; Connolly et al., 2014; Knoepfler, 2019d; Munsie et al., 2017; Ogbogu et al., 2018; Turner and Knoepfler, 2016). Based on online advertising, the unproven SCI market is estimated at US$2.4 billion impacting 60,000 patients annually (International Society Cell and Gene Therapy (ISCT), 2018) with documented clinics in Australia, Austria, Canada, China, Colombia, Costa Rica, Dominican Republic, Germany, India, Israel, Japan, Korea, Malaysia, Mexico, the Netherlands, New Zealand, Panama, Philippines, Portugal, Russia, Spain, Thailand, Turkey, UK, US, and Ukraine.

The target demographic for unproven SCIs are older adults who are avid seekers of information on stem cells and regenerative interventions (Smith et al., 2020). Patients receiving unproven SCIs have resulted in neoplastic, neurologic and cardiovascular complications, and infections, some of which have resulted in significant morbidity and mortality (Bauer et al., 2018). The overall global harm to patients is challenging to reasonably estimate as many of these clinics do not report side effects and complications. An analysis of patient lawsuits against clinics showed that, in addition to seeking recompense for physical injuries, plaintiffs sought compensation for the infliction of emotional distress and financial wrongs from unproven SCIs (Horner et al., 2018). The worldwide distribution of clinics marketing unproven SCIs also causes social harm by diminishing trust in government institutions, such as the US Food and Drug Administration (FDA) and European Medicines Agency (EMA), and reducing their ability to ensure the development of safe and effective regenerative interventions. The growth of the unproven SCI industry and the harms to patients and society strongly suggests that tackling this issue should become a major global health priority and on the public health agenda of every nation.

### The limits of intra-national public health efforts to combat unproven SCIs

Public health efforts within individual nations have resulted in modest effects at closing clinics and stifling the market. Taking a closer look at the US policy landscape illustrates how regulatory bodies in one nation have made considerable policy efforts to curb the marketing of unproven SCIs (PEW Charitable Trusts, 2019), but the industry continues to evolve and grow.

The growth of the US unproven SCI market was noted to be about 570 clinics in 2015–2016 (Turner and Knoepfler, 2016), about 715 in 2017–2018 (Turner, 2018), and in 2019 is estimated to be about 1,000 (Knoepfler, 2019d). In 2017, the FDA revised their guidance on cell-based therapies, which reiterated that all human cells, tissues, and cellular- and tissue-based products (HCT/Ps) that are more than minimally manipulated and/or intended for non-homologous uses will be regulated as a drug, device, and/or biologic (U.S. Food and Drug Administration, 2017a, 2017b). The new guidance also clarified definitions of key terms (i.e., homologous use) as well as provided examples of procedures. In cases where guidance has been insufficient to stop non-compliant practices, more aggressive means to shut down clinics have been undertaken. Based on the request of the FDA, the Department of Justice filed permanent injunctions against two stem cell businesses (PEW Charitable Trusts, 2019). In one case against a Florida clinic, a federal judge ruled for the FDA, indicating that the agency has the authority to regulate adipose-derived cells (Grady, 2019). While a win for the FDA could help the agency curb the unproven SCI market, such cases can take years to resolve. The process usually begins through initial enforcement actions, including inspections and warning letters, before seeking injunctions extending the time it takes before the clinic would discontinue operations. Moreover, the FDA's enforcement arm has limited capacity (PEW Charitable Trusts, 2019). Given that nearly 70% of clinics have one to three practitioners, of which 40% are solo practices in the US (Fu et al., 2019), many clinics may escape enforcement action because the FDA is unable to tackle every clinic advertising unproven SCIs. Therefore, it is unlikely that a single agency, such as the FDA, will be able to stop the industry effectively.

Also at the federal level, the US Federal Trade Commission (FTC) oversees false or misleading advertising and can limit SCI clinics. In 2018, the FTC announced that it settled charges with a California-based SCI clinic making deceptive claims (U.S. Federal Trade Commission, 2018). Nevertheless, due to its expansive scope, declining budget, and the small size of the agency, the FTC has limited ability to take considerable enforcement action (PEW Charitable Trusts, 2019).

At the state level, there has been sporadic activity by some state Attorneys General to target clinics and clinicians who offer unproven SCIs. In North Dakota, the Attorney General's office forced a SCI clinic to pay nearly $20,000 to refund patients as well as a civil penalty over concerns about misrepresentation and potential adverse events (Emerson, 2018). In 2019, the New York State Attorney General's office filed a lawsuit against a Manhattan clinic providing unproven adipose-derived SCIs (Knoepfler, 2019a). In 2020, the State Attorney General in Georgia filed suits against several clinics for false and misleading claims about unproven regenerative products where profits of $6.4 million were made through aggressive marketing to at least 842 consumers (Knoepfler, 2020; Office of the Attorney General, 2020).

There has also been some activity by state medical boards. The Federation of State Medical Boards published recommendations in 2018 and outlined that among the 51 state medical boards, 17 had investigated complaints against physicians related to unproven SCIs that resulted in 8 disciplinary actions (Fedration of State Medical Boards, 2018). The Medical Board of California formed a task force to address the high number of clinics offering unproven SCIs, but has yet to take actions against clinics or release a report addressing the state's market (Knoepfler, 2019b). A few states have introduced or enacted legislation requiring clinics to inform patients that the SCIs being offered are not FDA approved, or requires the registration of SCIs with the state (PEW Charitable Trusts, 2019). However, Texas, went the opposite direction, passing legislation in 2017 and 2019 that allows patients to access unregulated SCIs and protect physicians administering these interventions (Matthews et al., 2018). It remains unclear whether the Texas law conflicts with federal statute and FDA guidelines.

This examination of the US regulatory landscape implies that the one-off actions by some federal and state agencies and offices may be motivated by individual complaints, suggesting that greater coordination is needed. These efforts, however, have not gone unnoticed and new media narratives have shifted from medical cures toward the dangers of unproven SCIs (Beil, 2020). While several clinics closed shops in the US, some have spread to new locations in the Middle East, Caribbean, and Eastern Europe. Therefore, it became evident that a regulatory approach within each nation in isolation may not sufficiently address the spread and proliferation of unproven SCI clinics. There is also a necessity to assess the migration of clinics to countries with less-specific regulations around cell-based therapies.

### WHO's role in curbing global health risks

Global health policy, compared with national public health efforts, can incorporate international strategies and harmonization efforts that have a broader reach than any single country (Ruger and Yach, 2014). Although several organizations work on global health issues, WHO, as defined in its constitution, serves a coordinating function related to international public health (Ruger and Yach, 2014). WHO has the authority to establish collaborations; assist governments; propose conventions, regulations, and agreements; provide recommendations and technical guidance; and improve standards. It promotes cooperation among different nations, specialized agencies, scientific and professional groups, and assists in establishing a common set of norms and product development standards (Lee et al., 2017). It is the only global health organization where each nation state has one vote offering it a unique form of legitimacy when convening its authority over policy decisions on international health matters (Kickbusch and Hein, 2010). WHO is also entrusted to maintain effective collaboration with the United Nations and governmental and non-governmental health organizations, including the International Society for Stem Cell Research (ISSCR), FDA, and EMA among other organizations involved in addressing unproven SCIs. In many areas of global health, WHO has been particularly effective in coordinating responses to major global health threats, including communicable disease outbreaks (such as the COVID-19 pandemic) and humanitarian crises.

In 2018, WHO announced the establishment of an expert panel on human genome editing (World Health Organization, 2018b). Advancing technologies, in particular CRISPR-Cas9, permits gene editing with more accuracy and relative ease and reignites ethics and policy debates from the 1980s about gene transfer research (Cathomen et al., 2019; Meagher et al., 2020). The two major policy topics surrounded the permissibility of heritable germline (versus somatic) editing and the use of gene editing for enhancement (versus therapeutic) purposes. These discussions were brought to the forefront first in 2015 when a group in China edited a human embryo and later in 2018 when, unbeknown to the scientific community, He Jiankui implanted genetically modified human embryos, resulting in the birth of three children with heritable germline modification in the *CCR5* gene believed to confer HIV resistance in the babies. Such an undertaking caused public outcry and alarm within research and health practice communities. As a result, the WHO established an expert panel to develop global standards for governance and oversight of human genome editing (World Health Organization, 2019b).

The WHO Expert Advisory Committee (EAC) on Human Genome Editing was established with a mandate to examine the scientific, ethical, legal, and social challenges of somatic and germline human genome editing (World Health Organization, 2019b). The WHO EAC is charged with three activities: (1) to review the current literature on research involving human genome editing and its applications; (2) to consider existing proposals for governance and relevant ongoing initiatives; and (3) to solicit findings of public attitudes toward the uses of human genome editing (World Health Organization, 2019b). One of the major roles of the EAC on Human Genome Editing is to construct an ethics and policy framework. Toward this goal, the EAC has developed a draft framework grounded on ethical principles that can identify issues, ways to address them, and be applied to existing governance frameworks in any country (World Health Organization Expert Advisory Committee, 2020). Similar to the goals of the EAC on Human Genome Editing, a WHO EAC on Regenerative Medicine could also review existing proposals for governance and solicit information on public perceptions to inform normative and practice decisions.

### A WHO EAC on Regenerative Medicine

Multiple scientific, ethical, legal, and social issues impact the responsible translation of stem cells and regenerative medicine (Abou-El-Enein et al., 2015, 2014; Bauer et al., 2018). A WHO EAC on Regenerative Medicine could address several issues, including standardization of regulatory definitions and practices; the need for robust scientific data on the safety and efficacy of cell-based therapies balanced with patients' unmet medical needs; adequate protection of participants in first-in-human regenerative therapy trials; and informing patients and clinicians in an area of substantial misinformation (Chan, 2017; Lee et al., 2017; MacPherson and Kimmelman, 2019; Richardson et al., 2020; Sipp et al., 2017). A WHO expert panel should prioritize those topics related to the unproven SCI industry that create the most harm to patients and the regenerative medicine community. While it is beyond the scope of this article to go into depth about these issues, we briefly highlight the importance of a few contemporary topics.

Perhaps one of the most important topics the WHO EAC on Regenerative Medicine can address is the harmonization of regulatory definitions and practices for cell-based therapies. Regulations need unambiguous definitions of key concepts that are harmonized and adopted between countries so that clinics marketing unproven SCIs cannot escape regulatory oversight and relocate to a permissive environment. As mentioned earlier, the FDA’s 2017 revised guidelines pertaining to cell-based therapies clarified terms, such as minimal manipulation and homologous use (Table 1), while providing explicit examples of products and procedures that are regulated versus those that do not fall under regulatory oversight (Table 2). Similarly, the European Commission (Regulation (EC) no. 1394/2007) requires that any somatic cell therapy medicinal product (sCTMP) or tissue-engineered product that is manipulated and/or intended for non-homologous use (the transplanted HCT/P is used for a different function) falls under and is regulated as an advanced therapy medicinal product (Box 1; Table 1) (European Medicines Agency (EMA) Committee for Advanced Therapies (CAT), 2015; The European Commission, 2007). While the definitions and provisions among regulations between national agencies are not identical (see Table 1 for comparison), they still protect against more hazardous practices that lead to patient harm. Using existing policies and regulations as a model, the WHO EAC on Regenerative Medicine could develop a regulatory framework or template regulations for countries to adopt. Indeed, the development of a governance framework that could be adopted and applied to existing regulatory mechanisms would serve to provide ways to inform stakeholders and strengthen individual national regulations (Lee et al., 2017). This would allow industry and clinics to understand more fully what techniques and products will be regulated and what is considered outside the scope of regulatory oversight.

**Table 1:** Key concepts and definitions in the US FDA and EMA cell-based therapy regulations

General concept	US FDA terminology^a^	US FDA definition	EMA terminology^b^	EMA definition
Cell-based interventions	human cells, tissues, and cellular and tissue-based products (HCT/Ps)	products “containing or consisting of human cells or tissues that are intended for implantation, transplantation, infusion or transfer into a human recipient”	somatic cell therapy medicinal product	somatic cell therapy medicinal product is a biological product that contains or consists of cells/tissues that have been subjected to substantial manipulation or are not intended to be used for the same essential function in the recipient as the donor, but is intended to be used for therapeutic purposes
	HCT/Ps	see above	tissue-engineered products	a product contains or consisting of engineered cells and/or tissues, and is presented as having properties for, or is used in or administered to regenerate, repair or replace human tissue
Homologous use	homologous use	the cells have the same basic function(s) in the recipient as donor	same essential function	the cells when removed from their original environment in the human body are used to maintain the original function(s) in the same anatomical or histological environment
Autologous use	same surgical procedure	related to HCT/Ps, an establishment removes HCT/Ps from an individual and then implants, infuses, or transfers those cells/tissues into the same individual. Furthermore, the HCT/Ps are in their original form	N/A	
Manipulation	minimal manipulation	processing does not alter the relevant original/biological characteristics of the cell/tissue. For structural tissues, this includes its utility for reconstruction, repair, or replacement. Examples include rinsing, cleansing, sizing, or shaping	substantial manipulation	the cells/tissue have been manipulated during the manufacturing process so that their biological characteristics, physiological functions, or structural properties have been modified to be relevant for their intended function. Examples of substantial manipulation includes cell culture expansion, enzymatic digest, genetic modification of cells, and differentiation with growth factors. Examples of non-substantial manipulation include cutting, grinding, shaping, centrifugation, sterilization, irradiation, cell separation, filtering, and freezing
Structural tissue		HCT/Ps that physically support or serve as a barrier or conduit, or connect, cover, or cushion. Examples include bone, skin, amniotic fluid, umbilical cord, blood vessel, adipose tissue, cartilage, and tendon or ligament	N/A	
Nonstructural tissue		HCT/Ps that serve metabolic or other biochemical roles in the body are considered cells/nonstructural tissues. Examples include reproductive cells and tissues; hematopoietic stem/progenitor cells and lymph nodes	N/A	

N.A, not applicable—the concept was not described or was relevant to the policy.

a US FDA. 2017 “Regulatory Considerations for Human Cells, Tissues, and Cellular and Tissue-based Products” and “Same Surgical Procedure Exception under 21 CFR 1271.15(b)” (U.S. Food and Drug Administration, 2017a, U.S. Food and Drug Administration, 2017b).

b Regulation (EC) No. 1394/2007 and 2015 “Reflection paper on classification of advanced therapy medicinal products” (European Medicines Agency (EMA) Committee for Advanced Therapies (CAT), 2015; The European Commission, 2007).

**Table 2:** Procedures using adipose tissue and their regulatory status (homologous use, not regulated, or non-homologous use, regulated)

Example procedure^a^	Regulatory use
Adipose tissue is used to fill voids in the face or hands (e.g., for cosmetic reasons). This is homologous use because providing cushioning and support is a basic function of adipose tissue^b^	homologous use
An HCT/P from adipose tissue is used to treat musculoskeletal conditions, such as arthritis or tendonitis by regenerating or promoting the regeneration of articular cartilage or tendon	non-homologous use
An HCT/P from adipose tissue is used to treat neurological disorders, such as multiple sclerosis, by limiting the autoimmune reaction and promoting remyelinization	non-homologous use
Adipose tissue is used for transplantation into the subcutaneous areas of breast for reconstruction or augmentation procedures	homologous use

a US FDA. 2017 “Regulatory Considerations for Human Cells, Tissues, and Cellular and Tissue-based Products.” Example 19-6.

b The FDA defines the basic functions of adipose tissue as “providing cushioning and support for other tissues, including the skin and internal organs, storing energy in the form of lipids, and insulating the body.”

Box 1 **Elaboration on the regulatory definitions of cell therapy products**

The EMA’s 2007 regulation and 2015 reflection paper state that any somatic cell therapy medicinal product (sCTMP) or tissue-engineered product (TEP) that is manipulated and/or intended for non-homologous use (different essential function) falls under the category of advanced therapy medicinal product and regulated as such (for detailed definitions see Table 1) (European Medicines Agency (EMA) Committee for Advanced Therapies (CAT), 2015; The European Commission, 2007). The difference between sCTMP and TEP is related to the intent of the product—whether it is for treating, diagnosing or preventing diseases (sCTMP), or regenerating, replacing, or repairing tissues (TEP). Cells that are used for regeneration, replacement, or repair of tissue are classified as a TEP despite being cellular and not tissue based. The guidelines also explicitly describe the techniques considered minimal manipulation (e.g., centrifugation, microbial solutions, and sterilization) as well as what is considered substantial manipulation (e.g., cell expansion and enzymatic digestion) (Table 1).

The FDA’s 2017 guidance documents reiterate that all HCT/Ps that are more than minimally manipulated and/or intended for non-homologous uses will be regulated as a drug, device, and/or biologic (U.S. Food and Drug Administration, 2017a, 2017b). In addition, the guidance documents define key terms, including minimal manipulation, same surgical procedure, and homologous use (see Table 1). The FDA also separates tissues into two categories: structural versus nonstructural tissues. These categories allow the FDA to define tissue functions more specifically, which in turn linked with their requirement related to homologous use. With all of these details, the FDA includes examples to clarify the regulations to the industry. This includes giving examples of common structural tissues and nonstructural tissues (Table 1). They also walk you through how cells/tissues could be used for specific treatments using “same surgical method” or “minimal manipulation” exemptions as well as incidents where they cannot (see Table 2 for an example).

A second key issue that impacts patients, clinicians, and scientists is the need for robust clinical evidence during product development to offer safe and effective cell therapies to patients with unmet medical needs (Elsallab et al., 2020). Many among the scientific community have stressed the importance of conducting well-designed and scientifically rigorous clinical trials, most notably by the ISSCR, which developed and revised clinical guidelines for stem cell-based therapies (International Society for Stem Cell Research, 2016). Yet many patients have neither the time nor the desire to wait for full product development and some would be willing to try unproven SCIs with insufficient safety and efficacy data (Hawke et al., 2019; Petersen et al., 2014; Rachul, 2011) and assume the risks associated with them (Petersen et al., 2015). This tension between the desires of the scientific and patient communities is seen in various areas of health care where patients behave as active consumers of health services and want to be presented with options even if they are scientifically unsubstantiated and unapproved by a regulatory agency (Chan, 2017). Advocates argue that unproven SCIs should be permitted similar to investigational drugs offered through trials or compassionate use programs. Opponents, however, explain that the context is not similar. Investigational drugs in clinical trials, accelerated access programs, and FDA non-trial mechanisms, i.e., compassionate use programs, are provided under controlled conditions, with strict manufacturing standards and oversight. Even the more lenient US federal Right-to-Try law requires the intervention to have completed phase 1 safety testing. While these debates are often displayed as competing interests, the WHO EAC on Regenerative Medicine could deliberate to determine how best to balance these interests and show the underlying common goal: to create safe and effective cell therapies that improve the lives of patients. Such strategies may require developing methods for regulatory streamlining without reducing the scrutiny of the current regulatory review process, further investment into clinical research, and increasing access of patients to legitimate clinical trials, compassionate use programs, and the creation of novel ways to inform patients and physicians interested in such treatment options.

Finally, a WHO EAC on Regenerative Medicine could formulate an effective information campaign strategy about unproven SCIs for countries to adopt in an effort to correct misinformation about SCIs. Unproven SCI providers routinely use a range of sophisticated marketing strategies to showcase their products as scientifically legitimate, overemphasizing benefits, underplaying risks, and omitting information that could reduce the chances of a successful sale. Many of these clinics use fake scientific articles or point to animal studies to demonstrate treatment efficacy (Richey and Frow, 2019), seek dubious for-profit ethics boards for token approval (Knoepfler, 2019c), and list unproven SCIs as pay-to-participate trials on ClinicalTrials.gov since the registry is not monitored (Turner, 2017). A WHO EAC on Regenerative Medicine could provide education materials to better inform patients, mobilize support from patient advocacy groups, and perhaps even develop a global registry that will be monitored to contain legitimate clinical studies similar to that being considered by the WHO EAC on Human Genome Editing. While this proposal focuses on having WHO develop an EAC, other organizations that command respect and buy-in from the international community may also tackle the global health issue of unproven SCIs.

### Potential limitations

One limitation is that some may argue that the spread of clinics providing unproven SCIs is not an immediate or significant enough issue to be addressed by WHO, or any other international body, given the many other pressing global health calamities, including WHO's response to pandemics, which should take precedence. Prioritizing immediate health threats, however, does not negate the need to address the global health issue of non-communicable diseases, which has been well established as a leading cause of death around the world (World Health Organization, 2005, 2014, 2018a, 2018b; Wagner and Brath, 2012), and the impact the rogue unproven SCI industry may have on the advancement of legitimate regenerative medicine research. Moreover, taking appropriate action now to curb this industry would limit the ongoing exposure of patients to life-threatening complications and unnecessary deaths from unproven SCIs worldwide.

A second and related limitation is the reduced priority that may be given to addressing issues, such as the unproven SCI market during agenda setting by WHO if the US were to withdraw from the organization. In response to the COVID-19 pandemic, former President Trump withdrew US support of WHO in July 2020—a decision reversed in 2021 by President Biden (Gostin et al., 2020; Weintraub, 2021). The US and the Bill and Melinda Gates Foundation constituted 25% of the WHO's 2018–2019 financial contributions (World Health Organization, 2019c). Since, the priority setting of the agenda is influenced by many factors, including the importance of topics to target donors (People's Health Movement, Medact, Third World Network, Health Poverty Action, Medico International, 2017), the possible withdrawal of the US again may reduce the chances that the issue of the unproven SCI industry would be center attention for WHO and member states should the US delegation bring this topic forward. And, given that the US is also the largest funder for science and engineering R&D in the world and that its policies influence other countries (National Science Board, 2020), without its participation in WHO, recommendations and standards developed by WHO may be less likely to be implemented within the US and other countries. However, this limitation is hypothetical and assumes that the importance in addressing concerns about the unproven SCI industry does not achieve a high enough priority for WHO and other countries. It is noteworthy that most of the current 194 nation members of WHO are countries known to have clinics selling unproven SCIs (World Health Organization, 2021), suggesting that the topic, at minimum, affects the majority of member states.

A third limitation is that recommendations made by the WHO would have limited impact on member and non-member states. Although a key function of the WHO is to carry out international conventions and agreements, as well as implement regulations and non-binding standards and recommendations (Ruger and Yach, 2009), the WHO has no power to impose health policies on national governments, prioritize such efforts on national agendas, or announce sanctions. The organization acts as a consultant with the assumption that nation states will implement its recommendations. Recognition of the WHO's regulatory and political authority is necessary for its recommendations to be adopted by member states and implemented in corresponding legislative and other policy mechanisms (World Health Organization, 2006). While noting this limitation, many undertakings by the WHO have been successful on the international health stage and policies and frameworks have been widely adopted, including the eradication of smallpox, TB, and other infectious diseases (Henderson, 1987; Raviglione, 2003; Raviglione and Uplekar, 2006), and the Framework Convention on Tobacco Control (Ruger, 2005). Given these past successes, the tremendous health and social impact of unproven SCIs, and that most countries known to have SCI clinics are member states, it is likely that many countries will adopt, at least in part, a comprehensive framework developed by the WHO to address this international health problem. One test will be seeing how universally adopted the guidelines and recommendations for heritable germline editing are when released.

### Conclusions

The unproven SCI industry threatens the advancement of regenerative medicine. Reports of adverse events from unproven SCIs has the potential to affect funding and clinical trial recruitment, as well as increasing burdens among regulatory agencies to oversee the industry. Other examples of high-profile incidents have prompted a reexamination of regulatory frameworks and public health priorities, such as the death of Jesse Gelsinger in a notable gene therapy trial, or the more recent creation of germline-gene-edited children. Fortunately or unfortunately, the multiple ethical, social, and physical harms resulting from unproven SCIs have not garnered similar attention. However, it is impossible to speculate when outcry might raise sufficient awareness to prioritize the issue.

Permitting unregulated SCIs to flourish demonstrates a lack of concern over patient welfare and undermines the need for scientific evidence for medicinal product R&D. While some regulatory agencies have limited oversight or enforcement powers, or choose not to use them, unproven SCI clinics still serve to undermine authority given to regulatory agencies and may reduce public trust impacting the development of safe and effective therapies. Addressing the continued proliferation of clinics offering unproven SCIs is a problem worth addressing now.

Due to the global growth of clinics offering unproven SCIs, it is crucial to start placing this important issue on the agenda of international health organizations. Harmonizing with executive and judicial authorities to accelerate the implementation and execution of new regulations can save significant efforts to curb the operation of unauthorized businesses. Similar efforts could be initiated to address the spread of unproven SCIs and promote greater discussion on exerting more efforts toward harmonizing perspectives, educational and outreach tools, and potential policies. The spread of unproven SCIs globally reflects critical gaps in the international system for responding to health crises. Urgent measures are needed to address these gaps and enhance the global capacity to detect and respond to this eminent crisis. One tool that has not been used is the influence and connection of WHO to create an EAC on Regenerative Medicine that would provide guidance on public policy and public engagement.

## Author contributions

M.A. concieved the idea of having a global health solution address the unproven SCI industry. Z.M. and M.A equally drafted the first version of the manuscript. K.R.W.M. added new material surrounding regulatory definitions and the harmonization of regulations. All authors critically revised the manuscript for important intellectual content, responded to reviewers' comments, and agreed on the final version to be published.

## Conflicts of interests

The authors are Lawrence Goldstein Science Policy Fellows for the International Society for Stem Cell Research (ISSCR) and ex-officio members of ISSCR's Public Policy Committee. Z.M. is also a current member of ISSCR's Education Committee. The views expressed here are those of the authors and not of ISSCR or their respective academic institutions. None of the authors have received financial compensation from any organization for their work on this article.
